# Ferritin-based nanoparticle vaccine protects neonatal piglets against porcine epidemic diarrhea virus challenge following immunization of pregnant sows

**DOI:** 10.1186/s13567-025-01542-8

**Published:** 2025-07-07

**Authors:** Yangkun Liu, Jiaxin Deng, Zhen Bi, Mingzhan Luo, Xueying Han, Lunguang Yao

**Affiliations:** 1 Henan Provincal Engineering and Technology Center of Health Products for Livestock and Poultry, School of Life Science, Nanyang Normal University, Nanyang, 473061 Henan China; 2 College of Modern Service, Nanyang Vocational College of Science and Technology, Nanyang, 473061 Henan China

**Keywords:** PEDV, ferritin nanoparticle, lactogenic immunity, sows, piglets

## Abstract

Porcine epidemic diarrhea virus (PEDV) is an enteropathogenic coronavirus that causes severe diarrhea and high mortality in neonatal piglets, resulting in significant economic losses to the pig industry worldwide. Given that current commercial vaccines lack adequate protection against variant PEDV strains, there is an urgent need for the development of more effective vaccines to control PEDV infection. Here, a novel nanoparticle vaccine displaying the core neutralizing epitope CO-26 K-equivalent (COE) of PEDV spike protein was developed using *Helicobacter pylori* ferritin as an antigen delivery system, and its protective efficacy against the highly virulent PEDV LYL strain in suckling piglets was evaluated following immunization of pregnant sows. Compared to the COE monomer vaccines, COE-ferritin nanoparticle vaccines induced higher levels of PEDV-specific IgG, IgA, and neutralizing antibodies in the serum and colostrum of sows. Importantly, 3-day-old piglets born to COE-ferritin nanoparticle vaccinated sows were effectively protected against PEDV challenge, showing no clinical symptoms, less body weight loss, and rapid clearance of the virus in fecal swabs and intestines. Overall, our data demonstrate the protective efficacy of ferritin-based nanoparticles with the PEDV COE antigen, which may be further developed as a vaccine for sows to protect their piglets against PEDV.

**Supplementary Information:**

The online version contains supplementary material available at 10.1186/s13567-025-01542-8.

## Introduction

Porcine epidemic diarrhea (PED) is a highly contagious enteric disease characterized by severe diarrhea, vomiting, and dehydration, with a mortality rate of more than 90% in suckling piglets [1]. Since its first report in 1978, PED has spread to several European and Asian countries in the following decades; however, there were only sporadic and regional outbreaks until 2010 [2]. Since October 2010, large-scale PED outbreaks have occasionally occurred in many countries in Asia, North America and Europe, resulting in enormous economic losses to the swine industry worldwide [3, 4]. Unfortunately, most current conventional inactivated and attenuated vaccines are not sufficiently effective to control PEDV infection or have a potential biosafety risk [5, 6]. The development of an effective vaccine against PED is necessary to control the panzootic.

Porcine epidemic diarrhea virus (PEDV), the causative agent of PED, is an enveloped, single-stranded, positive-sense RNA virus belonging to the genus *Alphacoronavirus*. The PEDV genome is approximately 28 kb long and encodes four structural proteins [7]. The spike (S) protein located on the surface of the PEDV virion can induce the production of neutralizing antibodies because it harbors several neutralizing epitopes [8]. Among these neutralizing epitopes, the CO-26 K-equivalent epitope (COE) is responsible for recognizing and binding cellular targets, and is an important target of the host antibody response and regarded as the primary target for subunit vaccine development against PEDV infection. Previous studies have shown that the COE protein expressed by plants [9–11], *Lactobacillus* [12], *Bacillus subtilis* [13], and *Escherichia coli* [14] can be used to develop subunit vaccines in neonatal piglets. These vaccines can induce effective humoral immunity against PEDV, but failed to successfully cease the epidemic of PEDV. Therefore, more effective vaccine candidates are urgently required to prevent PEDV infection.

Owing to the ability to display multivalent antigens on the surface and generate enhanced immune responses, nanoparticles have emerged as an attractive platform in the field of vaccine development. As a type of nanoparticle, ferritin consists of 24 subunit proteins that self-assemble into a hollow spherical nanocage structure that is characteristic of ferritin nanoparticles [15]. Because they possess the characteristics of easily triggering innate immune responses, excellent thermal and chemical stability, and good biocompatibility, ferritin nanoparticles have great potential in vaccine development [16]. Ferritin nanoparticle-based vaccine candidates have been developed against several viral pathogens, such as severe acute respiratory syndrome coronavirus 2 [17], hepatitis C virus [18], human enterovirus 71 [19], human respiratory syncytial virus [20], and Zika virus [21].

In this study, a ferritin nanoparticle displaying the PEDV COE antigen as a vaccine candidate was developed. After immunization of pregnant sows, the immunogenicity was evaluated, and then the protective effect against PEDV transferred from immunized sows to their suckling piglets was assessed. Our study provides a novel strategy for developing a nanoparticle vaccine against PEDV infection.

## Materials and methods

### Cells, viruses, and antibodies

Vero cells were grown in Dulbecco’s Modified Eagle’s Medium (DMEM; Gibco, CA, USA) supplemented with 10% fetal bovine serum (FBS; Gibco, CA, USA) in a humidified incubator at 37 ℃ and 5% CO_2_. PEDV strain LYL (GenBank: ON960076) was isolated from piglet samples infected with PEDV and grown in DMEM containing 10 μg/mL trypsin. Mouse anti-PEDV COE polyclonal antibodies, which were obtained in a previous study, were stored at our laboratory [22].

### Plasmid construction

The COE-ferritin fusion gene was generated by fusing the COE gene derived from the PEDV LYL strain (GenBank: ON960076; nucleotide positions 22137–22555) to *Helicobacter pylori* ferritin (GenBank: NP_223316; residues 5–167) with a Ser-Gly-Gly linker and synthesized by Tsingke Biotech Co. Ltd (Zhengzhou, China). The fusion gene was codon-optimized prior to gene synthesis for efficient expression in *E. coli* and ligated into the pET28a vector using the *Nde*I and *Xho*I sites to construct the recombinant plasmid pET28a-COE-ferritin. The COE-coding region was separately PCR-amplified and cloned using the same restriction sites as described earlier in the pET28a vector for expression of the COE protein.

### Expression and purification of recombinant proteins

The expression plasmids pET28a-COE and pET28a-COE-ferritin were transformed into *E. coli* BL21 (DE3) competent cells. A single colony was picked and cultured in Luria–Bertani medium supplemented with 50 μg/mL kanamycin at 37 °C. When the optical density at 600 nm (OD_600_) of the culture reached 0.6–0.8, the protein expression was induced by adding 1 mM isopropyl-β-D-1-thiogalactopyranoside. After 6 h of induction at 37 °C, bacteria expressing the recombinant proteins were ultrasonicated, and aliquots of the supernatant and precipitate were analyzed using 12% sodium dodecyl sulfate polyacrylamide gel electrophoresis (SDS-PAGE). Subsequently, the recombinant proteins expressed as inclusion bodies were purified using Ni–NTA affinity chromatography columns (Qiagen, Sacramento, CA, USA), according to the manufacturer’s instructions. To assemble the COE-ferritin protein into spherical nanostructures, the purified protein was refolded in dialysis buffer (20 mM Tris–HCl, pH8.0, 10% sucrose, 0.6 mM Arg, 0.2 mM EDTA) with a gradually decreased urea concentration (6 M, 2 M, 0.5 M, and ddH_2_O). Finally, the protein samples were quantified using a BCA protein quantification kit (Sigma, St. Louis, MO, USA), and stored at −80 °C for further analysis.

### SDS-PAGE and western blot analyses

The purified protein samples were subjected to 12% SDS-PAGE and transferred onto polyvinylidene fluoride (PVDF) membranes that were blocked with 5% skim milk in TBST (50 mM Tris–HCl, pH7.4, 150 mM NaCl, and 0.05% Tween-20) at 4 °C overnight. After washing with TBST, the membrane was incubated with mouse anti-PEDV COE polyclonal antibodies (diluted 1:500) followed by HRP-conjugated goat anti-mouse IgG (H + L) (Boster, Wuhan, China). The membrane was incubated with a western blot substrate (biosharp, Beijing, China), and images were captured using a luminescent imaging system (Cytiva, Uppsala, Sweden).

### Transmission electron microscopy

The purified COE and COE-ferritin proteins at the concentration of 0.1 mg/mL were adsorbed onto a carbon-coated grid for 2 min. After washing with PBS, the grid was negatively stained with 2% phosphotungstic acid (pH 6.45) and observed using transmission electron microscopy (TEM; Hitachi H-7600) at 80 kV.

### Dynamic light scattering

The size of the COE-ferritin nanoparticles was characterized using dynamic light scattering (DLS), as previously described [23]. Briefly, purified COE-ferritin nanoparticles were diluted with PBS to a final concentration of 0.1–0.5 mg/mL. The size distribution of the nanoparticles was determined using a Malvern Zetasizer Nano ZS (Malvern Instruments Ltd., Worcestershire, UK), following the manufacturer’s protocol.

### Immunization and challenge experiment

Nine primiparous sows were randomly divided into three groups (*n* = 3; COE, COE-ferritin, and PBS groups), and each group was housed separately in a biosafety level 2 facility for the duration of the study. Sows in the COE and COE-ferritin groups were immunized at the fossa between the anus and tail base with either COE-ferritin nanoparticles (50 μg per dose) or COE protein (50 μg per dose) with a 50% (v/v) mixture of aluminium hydroxide adjuvant (Sigma, St.Louis, MO, USA). Three sows were immunized with PBS containing a 50% (v/v) mixture of aluminum adjuvant by the same immunization pathways. All sows were immunized twice at 4 and 2 weeks antepartum. Serum was collected from each sow at 0, 7, 14, 21, 28, 31, and 38 days post-immunization (dpi). At parturition, sows farrowed naturally, and colostrum samples from each sow were collected at 0 and 3 days after farrowing.

After being allowed to suckle for 3 days, three newborn piglets were randomly selected from each sow and challenged orally with a 1.0 mL dose of 10^5^ TCID_50_ of PEDV LYL virus. All piglets were monitored daily for clinical signs, fecal condition, body weight, and mortality from days 0 to 7 days post-challenge (dpc). Fecal consistency was scored as follows: 0 = normal; 1 = pasty; 2 = semiliquid; and 3 = liquid [22]. At 7 dpc, all surviving piglets were euthanized using sodium pentobarbital (50 mg/kg body weight) by an intracardial injection. Piglets were immediately necropsied either on death or euthanasia, during which intestinal tissue samples were collected for pathological evaluation and quantification of PEDV RNA load, respectively.

### Indirect ELISA for detection of PEDV‑specific IgG and IgA antibody

PEDV-specific IgG and IgA antibody responses were determined in serum and colostrum samples using an indirect ELISA. Briefly, the 96-well microplates were coated with 100 µL of PEDV LYL (10^5^ TCID_50_/mL) and blocked with 5% skim milk. Subsequently, the diluted serum or colostrum samples (1:100 in blocking buffer) were applied to the plate and incubated at 37 °C for 1 h. After three washes with PBST, HRP- conjugated goat anti-pig IgG (ab6915) or IgA (ab112746) antibody (Abcam, Cambridge, UK) was added and incubated at 37 °C for 1 h, followed by the addition of 3,3’,5,5’-tetramethylbenzidine substrate and incubation at 37 °C for 15 min. Finally, the reaction was stopped with 2 N sulfuric acid, and the absorbance was measured at 450 nm.

### Virus neutralization test

PEDV-specific neutralizing antibody (NAb) titers in the colostrum of sows and the serum of their offspring were determined using a virus neutralization test (VNT). Briefly, two-fold serially diluted samples were mixed with 200 TCID_50_ of PEDV LYL in equal volumes and incubated at 37 °C for 1 h. The mixture was then transferred to Vero cell monolayers in 96-well plates and incubated at 37 °C for 1 h. Following five washes with PBS, the cells were incubated in a maintenance medium supplemented with trypsin (10 μg/mL) at 37 °C with 5% CO_2_ for 5 days, with daily examination for cytopathic effect under an inverted microscope. The NAb titer was expressed as the reciprocal of the highest serum dilution that protected more than 50% of the cells from CPE, and calculated according to the Reed-Muench method.

### Cytokine assay

IFN-γ in serum samples collected from piglets was measured using a commercial pig IFN-γ ELISA kit (Ziker, Shenzhen, China), according to the manufacturer’s instructions. The concentration of each sample was calculated using a linear regression equation developed from serial standards.

### Quantitative reverse transcription polymerase chain reaction (qRT-PCR)

Each rectal swab was diluted and homogenized in 1 mL of sterile PBS and then centrifuged at 5000 × *g* at 4 °C for 10 min. An aliquot (200 μL) of supernatant was collected for viral RNA extraction. Equal quantities (0.1 g) of tissue samples from the duodenum, jejunum, ileum, cecum, colon, and rectum were homogenized in 1 mL of sterile PBS, and 200 μL of the supernatant was used for RNA extraction. The viral RNA was detected with qRT-PCR using specific primers and probe (PEDV-N-F: 5′-GGGTATTGGAGAAAATCCTGATAG-3′; PEDV-N-R: 5′-AACTGGCGATCTGAG CATAG-3′; and PEDV-N-Probes: 5′-AAGCAAC AACAGAAGCCTAAGCAG-3′), as previously described [12]. The viral copy number was calculated from a standard curve prepared using tenfold serial dilutions of known quantity of plasmid containing the PEDV *N* gene, plotting CT value versus RNA copy numbers.

### Histopathology and immunohistochemistry

At necropsy, the jejunum tissues were collected and fixed with 4% paraformaldehyde. The fixed jejunum tissues were embedded in paraffin, sectioned, mounted on slides, and stained with hematoxylin and eosin for histopathological examination. For measuring the villous height (VH) and crypt depth (CD), 10 different sites in each sample were randomly picked out and the average ratios of VH and CD were calculated. Intestinal lesion scores ranged from 1 to 4, with 1 being normal (VH:CD ≥ 3), 2 being mild villus atrophy (2 < VH:CD < 3), and 3 being moderate villus (1 < VH:CD < 2), 4 being severe villus atrophy (VH:CD < 1 or without villi or crypt). For immunochemistry (IHC), PEDV N-specific monoclonal antibody was used as the primary antibody, and HRP-conjugated goat anti-mouse IgG (H + L) was used as the secondary antibody.

### Ethics statement

All animal experiments were conducted under the approval from the Animal Welfare and Ethics Committee of Nanyang Normal University, permit number NYNU-2024–008, according to the National Guidelines on Animal Work in China.

### Statistical analysis

Statistical analyses were performed using GraphPad Prism 5 software (GraphPad Software Inc., San Diego, CA, USA). All data were represented as the means ± standard error of the mean (SEM). Statistical significance between different groups was determined using one-way analysis of variance (ANOVA), followed by Tukey’s multiple-comparison test. Statistically significant differences are denoted at the level of *P < 0.05 and **P < 0.01.

## Results

### Expression and purification of COE and COE-ferritin proteins

**Figure 1:**
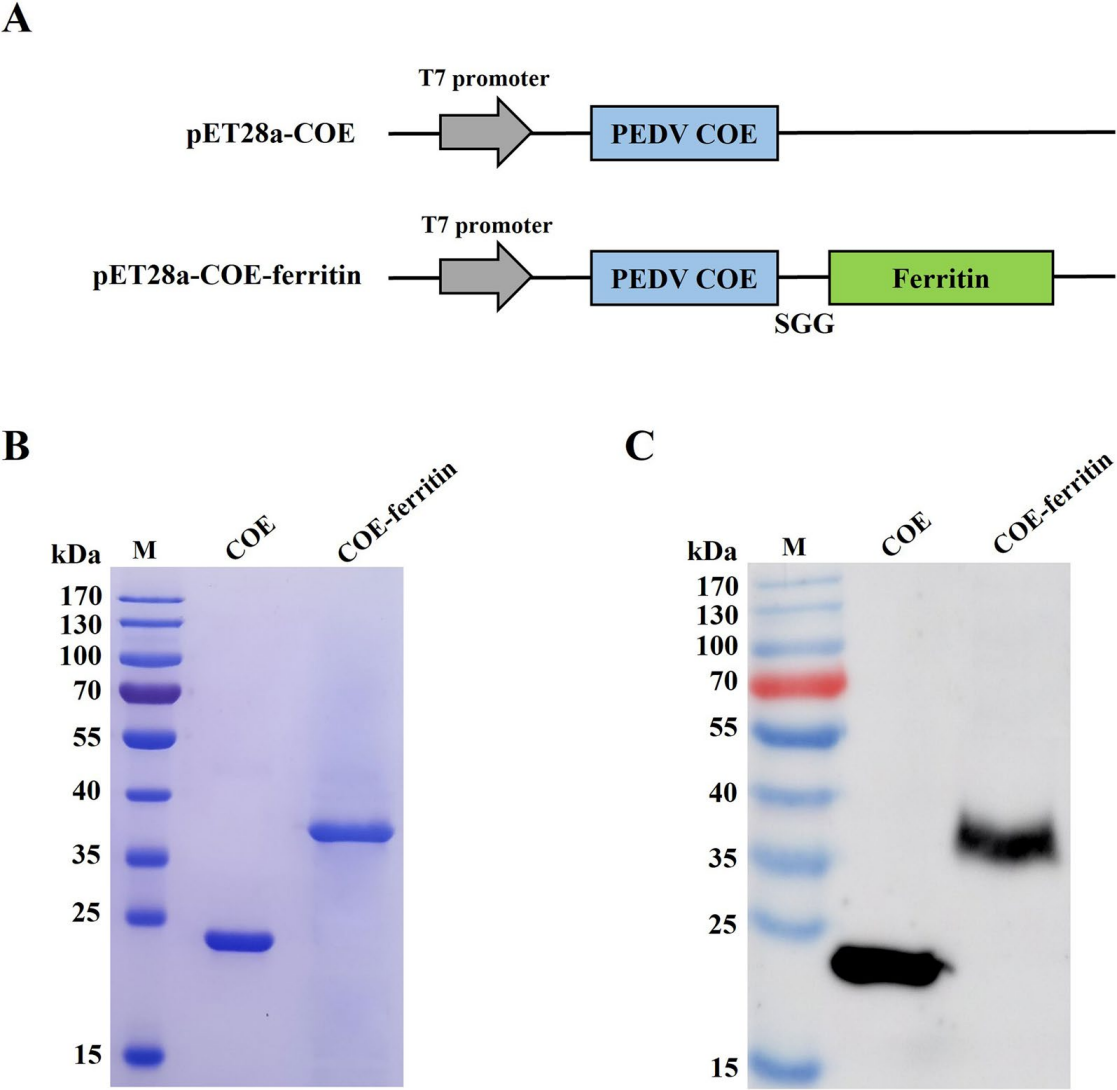
**Construction and purification of PEDV COE and COE-ferritin proteins.** **A** Schematic design of COE and COE-ferritin proteins. **B** Purification of COE and COE-ferritin proteins analyzed using SDS-PAGE. **C** Purification of COE and COE-ferritin proteins analyzed using western blot. Mouse anti-PEDV COE polyclonal antibodies and HRP-conjugated goat anti-mouse IgG (H + L) were used as primary antibodies and secondary antibodies, respectively.

For the expression of COE and COE-ferritin proteins, the prokaryotic plasmids pET28a-COE and pET28a-COE-ferritin were constructed (Figure 1A) and expressed in *E. coli* BL21 (DE3). After sonication and centrifugation, the supernatant and precipitate were collected and analyzed using SDS-PAGE. The results showed that both COE and COE-ferritin proteins were successfully expressed in *E. coli*, and the proteins were expressed mainly in the form of inclusion bodies (Additional file 1). As expected, the molecular weights of purified recombinant COE, along with COE-ferritin were approximately 22, and 38 kDa, respectively, as determined by SDS-PAGE analysis (Figure 1B). Simultaneously, western blotting revealed that COE and COE-ferritin specifically reacted with mouse anti-PEDV COE polyclonal antibodies (Figure 1C), suggesting that the purified proteins retained their antigenic properties. The yields of purified proteins, determined using the BCA protein quantification kit, were approximately 2.36 mg/L for COE and 2.17 mg/L for COE-ferritin.

### TEM observation of COE and COE-ferritin proteins

**Figure 2:**
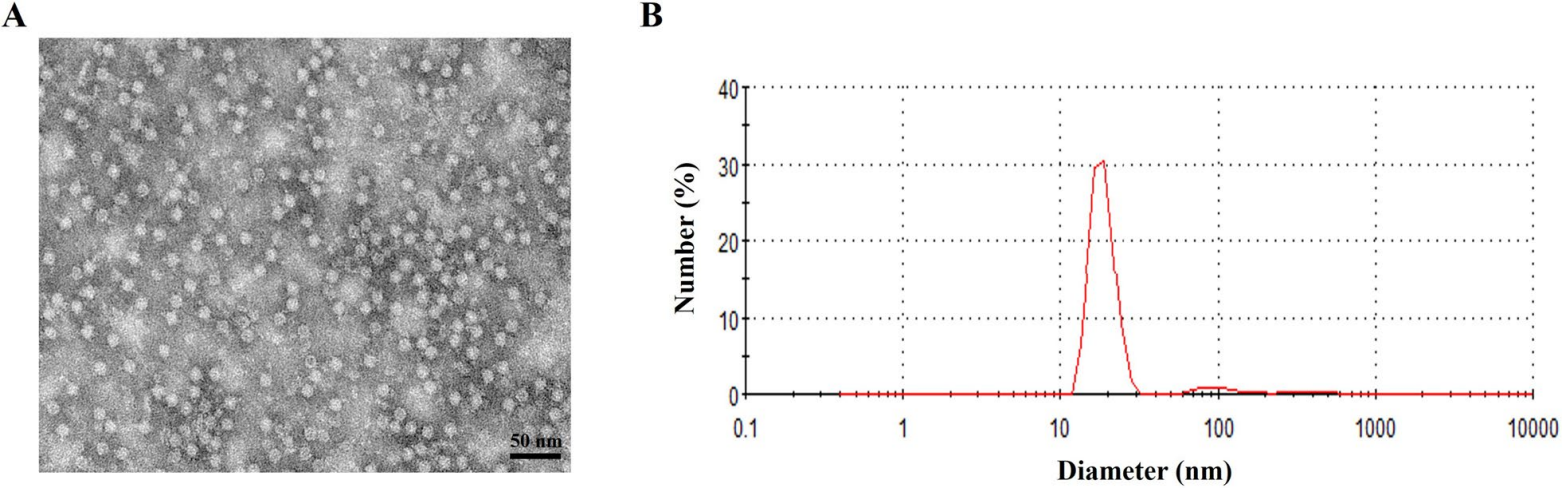
**Characterization of COE-ferritin nanoparticles.** **A** TEM images of the COE-ferritin nanoparticles; scale bar: 50 nm. **B** DLS analysis of the COE-ferritin nanoparticles.

To demonstrate nanoparticle formation, COE and COE-ferritin proteins were refolded via dialysis, as observed using TEM. The results showed that the purified COE-ferritin protein could be assembled into spherical nanoparticle structures of relatively uniform size (Figure 2A), whereas a similar structure was not found in the COE protein (Additional file 2). Consistently, the DLS results revealed that the average diameter of COE-ferritin nanoparticles was approximately 12 nm (Figure 2B). The results indicated that the COE-ferritin protein expressed in *E. coli* was able to form spherical nanoparticles.

### COE-ferritin nanoparticles induce potent antibody responses in sows

**Figure 3:**
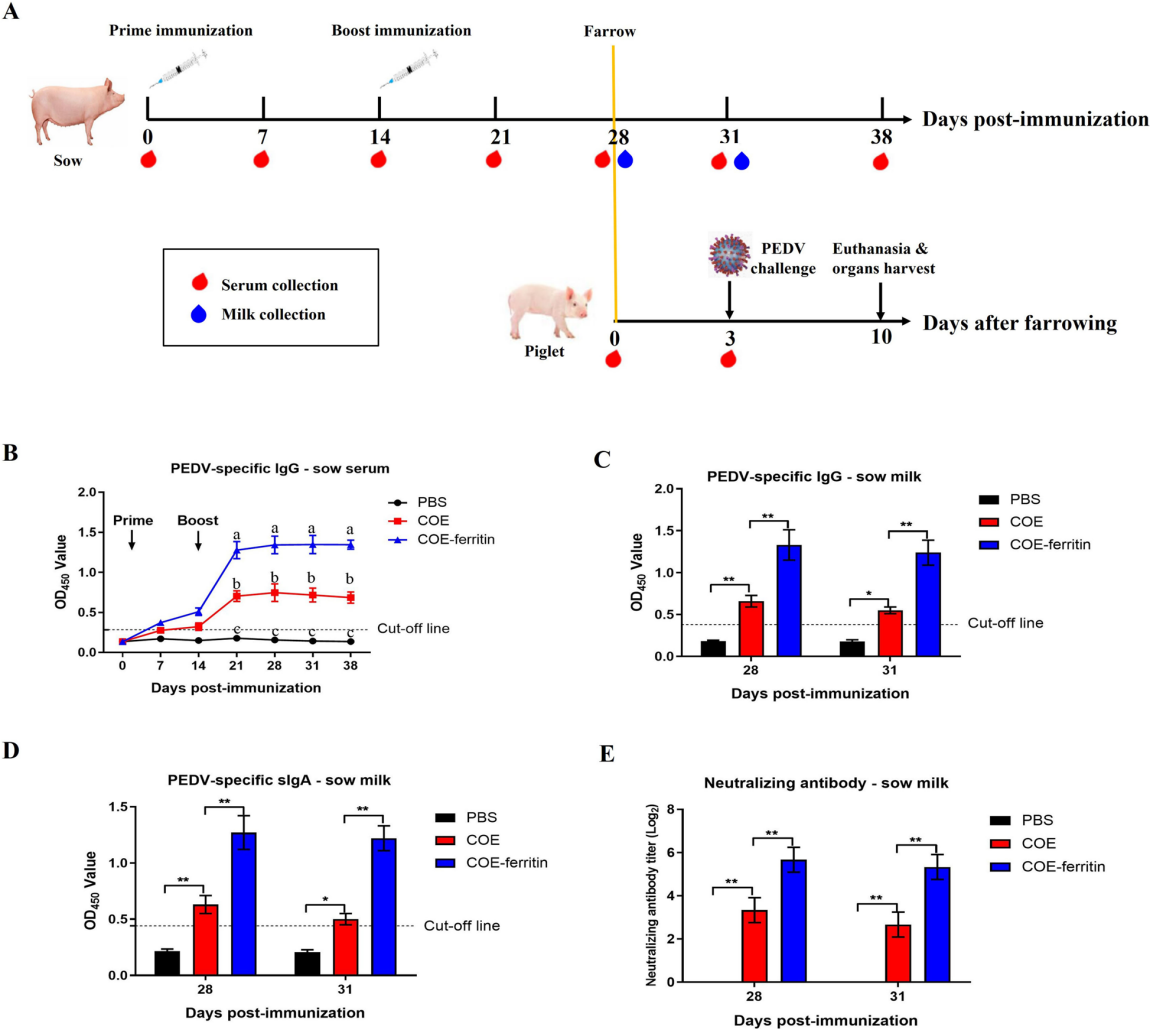
**Immunization of pregnant sows with COE-ferritin nanoparticles.** **A** Schematic representation of experimental protocol. Pregnant sows were immunized 28 and 14 days before farrowing with COE-ferritin, COE, or PBS. Serum samples were collected every week after the first immunization, and colostrum was collected 0 and 3 days after the farrowing day. All piglets were challenged orally with PEDV LYL 3 days after the farrowing day. **B** Detection of PEDV-specific IgG antibodies in sow serum using ELISA. There is a significant difference in the average value of different lowercased letters (*P* < 0.05). **C** Detection of PEDV-specific IgG antibodies in sow milk using ELISA. **D** Detection of PEDV-specific IgA antibodies in sow milk using ELISA. **E** NAbs titers in sow milk against PEDV LYL. Results are representative of three independent samples and presented as mean ± SEM (*P < 0.05, **P < 0.01).

Pregnant sows were immunized twice with COE, COE-ferritin, or PBS at 4 and 2 weeks antepartum to assess the immunogenicity of the nanoparticle vaccines (Figure 3A). Serum and milk samples were collected to evaluate antibody responses. As shown in Figure 3B, PEDV-specific IgG antibodies were detected at 14 dpi in both COE and COE-ferritin groups, and the antibody levels in the COE-ferritin group were significantly higher than those in the COE group at 21, 28, 31, and 38 dpi (*P* < 0.05). In addition, the levels of PEDV-specific IgG antibodies in the COE group gradually declined 28 days after the first immunization, whereas those in the COE-ferritin group remained high at 38 dpi. The level of anti-PEDV antibodies were detected in the sow colostrum/milk at 28, and 31 dpi. PEDV-specific IgG (Figure 3C) and sIgA (Figure 3D) antibodies, as well as neutralizing antibodies against PEDV LYL (Figure 3E) were detected in the colostrum/milk of immunized sows but not in those in PBS group, with higher levels in the COE-ferritin group than in the COE group.

### COE-ferritin nanoparticles confer passive immunity to piglets

**Figure 4:**
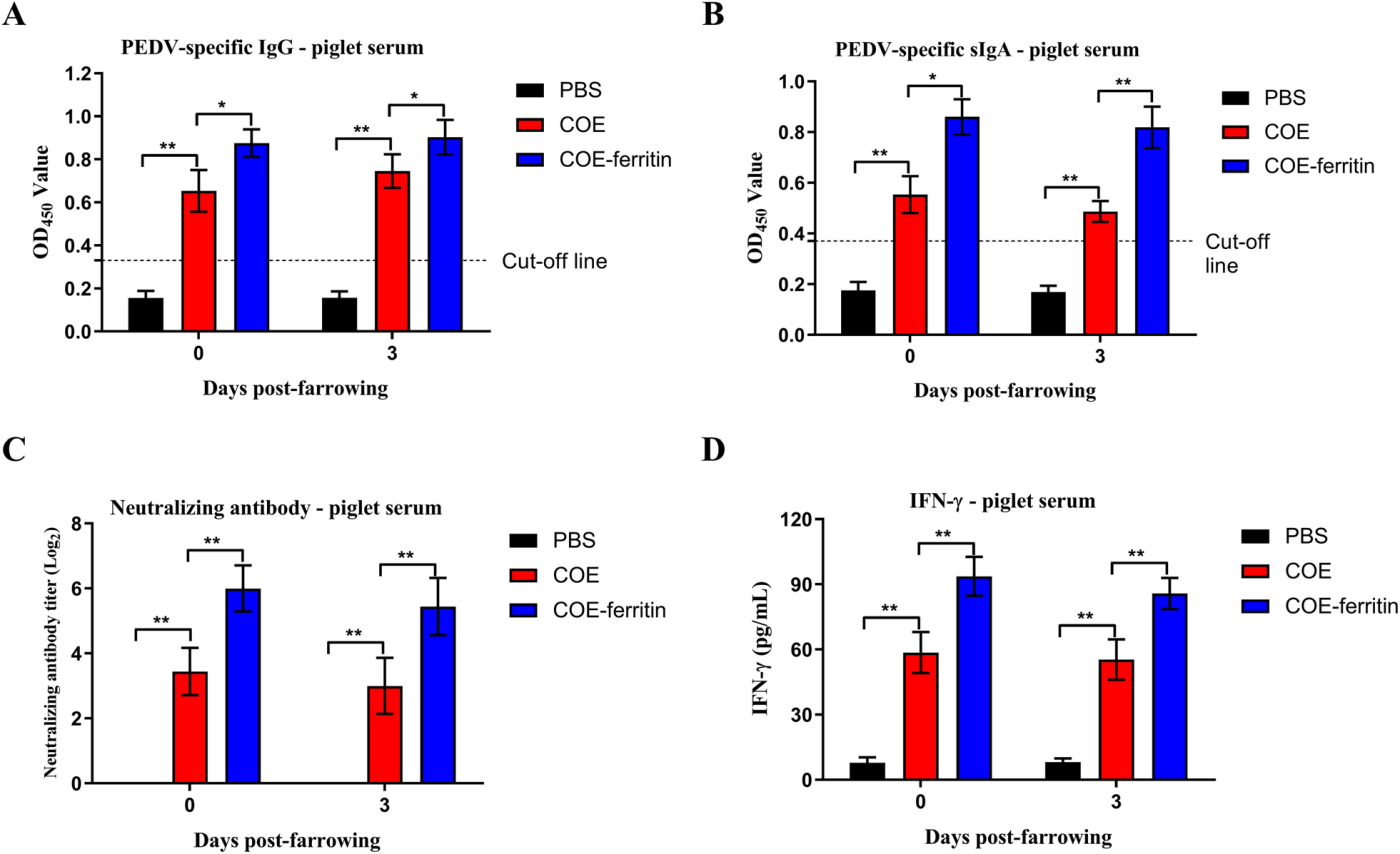
**Immune responses to COE-ferritin nanoparticles in piglets.** **A** PEDV- specific IgG antibodies in piglet serum were detected using ELISA. **B** PEDV-specific IgA antibodies in piglet serum were detected using ELISA. **C** NAbs titers in piglet serum against PEDV LYL. **D** IFN-γ concentration in piglet serum was determined based on the standard curve of the recombinant porcine IFN-γ standard in a commercial ELISA kit. Results are representative of nine independent samples and presented as mean ± SEM (*P < 0.05, **P < 0.01).

The passive transfer of antibodies from vaccinated sows to their offspring was detected using ELISA and VNT assays performed on serum samples collected from piglets before and after colostrum ingestion. No PEDV-specific antibodies were detected in any of the newborn piglets on the farrowing day prior to colostrum ingestion (Figure 4). High levels of PEDV-specific IgG and IgA antibodies were detected in the serum of piglets born to COE- and COE-ferritin-vaccinated sows 3 days post-farrowing, and both IgG and IgA levels were significantly higher in piglets born to sows vaccinated with COE-ferritin nanoparticles than in piglets born to sows vaccinated with COE (*P* < 0.01; Figure 4A and B). Additionally, these specific antibodies conferred virus neutralization (Figure 4C). In contrast, these antibodies were not detectable in piglets born to the non-vaccinated control sows.

To further characterize the cellular immune responses, levels of IFN-γ were quantified using ELISA. The results showed that the serum of piglets born to the sows vaccinated with the COE-ferritin generated significantly higher levels of IFN-γ compared to the COE immunized sows (Figure 4D). These results indicate that passive transfer of PEDV-specific IgG, sIgA and NAb antibodies, as well as IFN-γ responses occurs between vaccinated pregnant sows and their offspring through ingestion of colostrum.

### COE-ferritin nanoparticles protect piglets against PEDV virus infection

**Figure 5:**
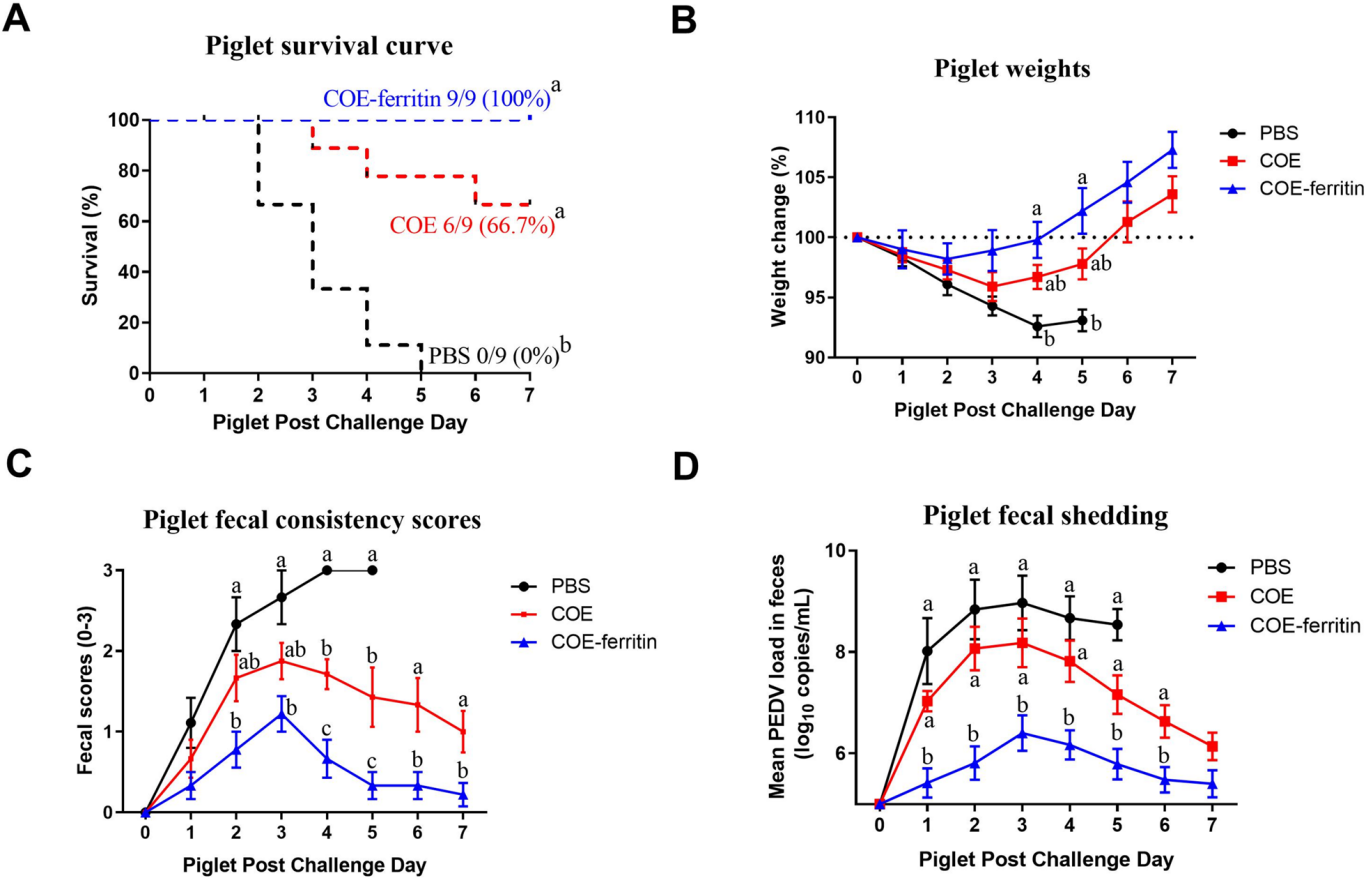
**Evaluation of the protective efficacy of COE-ferritin nanoparticles in piglets.** **A** Survival rate of piglets. **B** Body weight changes in piglets. **C** Fecal scores of piglets. **D** PEDV virus load in fecal swabs as determined by qRT-PCR. Each point represents the mean titer of the PEDV N gene in 1 mL of fecal swab sample from each group collected daily. There is a significant difference in the average value of different lowercased letters (*P* < 0.05).

To determine the protective efficacy of nanoparticles against PEDV, piglets born to either vaccinated or control sows were challenged orally with 1.0 × 10^5^ TCID_50_ PEDV LYL at 3 days of age. The survival and body weight of the piglets were monitored for 7 days after the challenge. All piglets (9/9; 100%) in the PBS group died by 5 dpc, whereas only three piglets (3/9; 33.3%) in the COE group died by 7 dpc, and none of the piglets (0/9; 0%) in the COE-ferritin group died after the PEDV challenge (Figure 5A). Piglet weight gain comparison showed that piglets in the COE-ferritin group had slightly higher normalized weights than those in the COE group (Figure 5B). Additionally, piglets in the PBS group had the lowest normalized weights throughout the study.

Fecal swabs were collected before and after the challenge to determine fecal consistency and viral RNA. The PBS group exhibited watery diarrheic feces at 2 dpc, and thereafter displayed typical clinical symptoms, including lethargy, loss of appetite, watery diarrhea, vomiting, and severe weight loss. Meanwhile, piglets in the COE-ferritin group began to have mild diarrhea at 2 dpc, and the average fecal scores of piglets in the COE-ferritin group were lower than those of piglets in the other groups (Figure 5C). PEDV RNA was detected in fecal swab samples from all challenged piglets, peaking at 3 dpc. Notably, PEDV RNA copy numbers were significantly lower in the COE-ferritin group than in the other groups throughout the experiment (*P* < 0.01) (Figure 5D). These results indicate that COE-ferritin nanoparticles provide strong protective immunity to relieve PEDV-induced clinical symptoms and accelerate viral clearance.

### COE-ferritin nanoparticles reduce small intestine damage post-PEDV challenge

**Figure 6:**
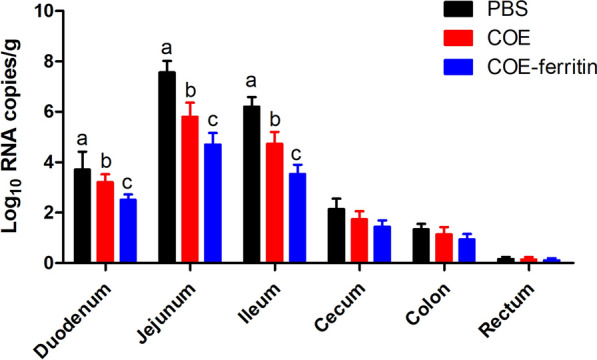
**PEDV distribution in different intestinal sites.** Data represents the mean ± SEM for nine piglets per group. Each column represents the mean titer of the PEDV N gene in 1 g the indicated intestinal segments in different groups at necropsy. There is a significant difference in the average value of different lowercased letters (*P* < 0.05).

The distribution of PEDV LYL in different intestinal tissues was detected using qRT-PCR after necropsy. All intestinal tissues from challenged piglets, including duodenum, jejunum, ileum, cecum, colon, and rectum, had PEDV distribution, and the highest viral RNA was detected in the jejunum (Figure 6). Moreover, the levels were significantly lower in the COE-ferritin group than in the other groups.

**Figure 7:**
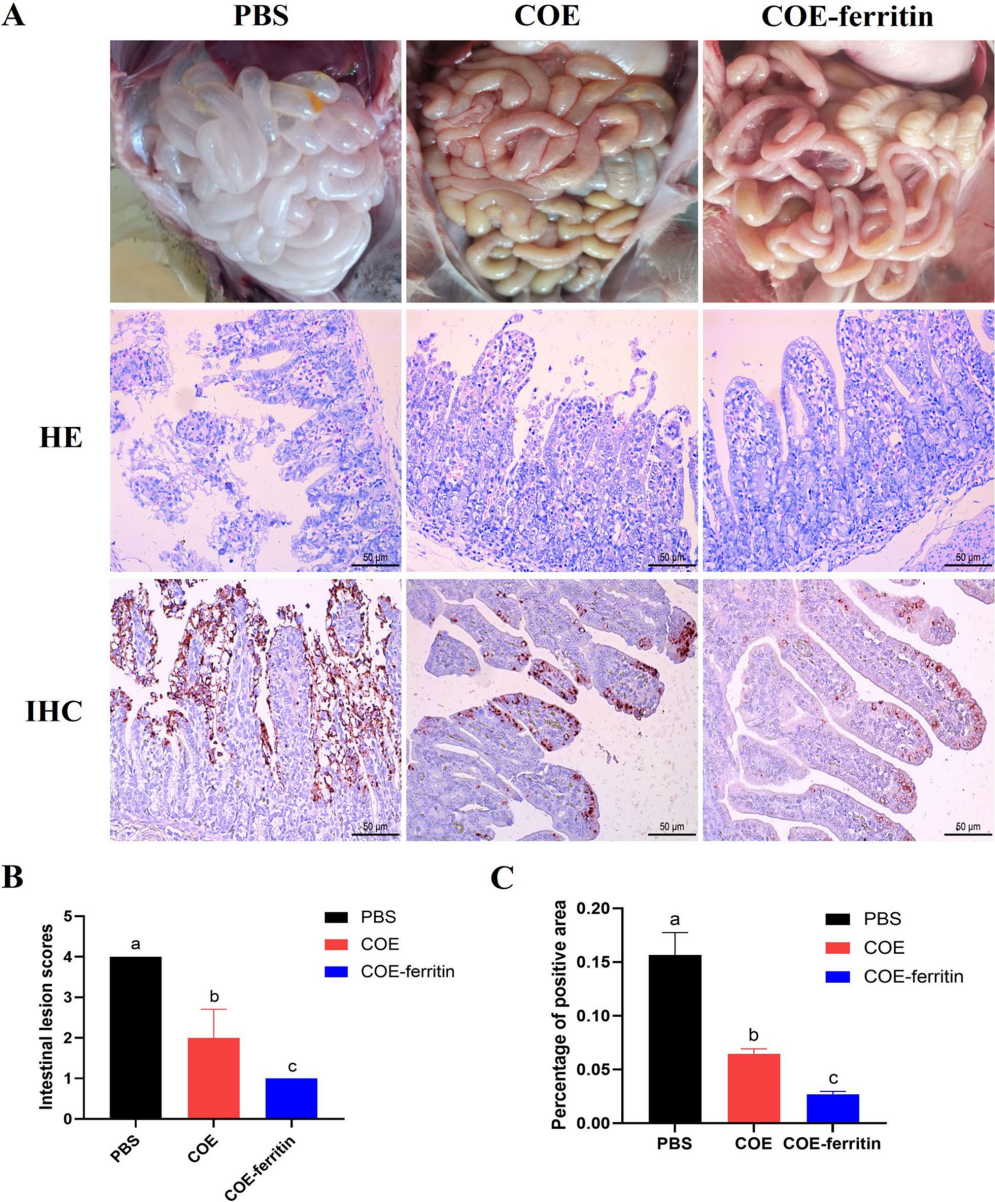
**Pathological response to the COE and COE-ferritin vaccines in suckling piglets.** **A** Gross pathology, histopathology and IHC analysis of intestines from PEDV challenge piglets. A representative intestine from three groups of piglets collected at necropsy, and jejunum tissue samples were collected and utilized for histopathological and IHC analysis. **B** Intestinal histopathological lesion scores. **C** The percentage of positive area detected by immunohistochemistry. There is a significant difference in the average value of different lowercased letters (*P* < 0.05).

Gross and histological lesions were evaluated in challenged piglets at necropsy. Typical PEDV lesions, such as the small intestine became thin-walled and gas-distended, were observed in piglets in the PBS group, while no obvious intestinal lesions were observed in piglets in the COE and COE-ferritin groups. Histopathological analysis showed that PEDV challenge induced obvious microscopic changes in jejunum tissues, which were characterized by villous atrophy, vacuolation, and loss of intestinal crypts, whereas the jejunum of COE- and COE-ferritin-vaccinated piglets showed no obvious pathological changes (Figure 7A). For the intestine lesion score, the average of VH:CD in the jejunum tissues from the COE-ferritin-vaccinated group were more than 3, which were significantly higher than those from COE-vaccinated group and PBS group (Figure 7B). Consistent with the histopathological results, IHC assays against PEDV N proteins revealed that the PEDV antigen was distributed mainly in the cytoplasm of atrophic villous epithelial cells and that the percentage of PEDV N-positive cells was significantly lower in the COE-ferritin-vaccinated group than in the other groups (Figure 7C). Taken together, these results indicate that the COE-ferritin nanoparticle vaccine confers newborn piglets with effective passive immunity, which protects them against virulent PEDV.

## Discussion

Since its discovery in southern China in October 2010, the highly virulent PEDV variant has spread rapidly in the pig industry worldwide, resulting in severe economic losses [25]. While several inactivated and attenuated vaccines have been developed and applied widely in the market, PED outbreaks with an 80–100% piglet mortality rate still occur in vaccinated herds, indicating that current vaccines cannot provide effective immune protective responses against epidemic strains [3, 26, 27]. Therefore, there is a constant need for alternative vaccine approaches against PEDV.

PEDV carries a highly glycosylated spike protein, which plays an important role in facilitating host cell attachment and entry [28]. The COE of the spike protein was confirmed to induce the production of neutralizing antibodies and, thus, is an ideal target for vaccine development [9–14]. Despite tremendous efforts to develop COE-based vaccines, the application of the COE protein as a vaccine candidate is still hindered by its low immunogenicity. To overcome this limitation, various approaches have been used to enhance the immune response to the COE antigen [14, 29, 30]. One of the most promising approaches is to display COE antigen domain in self-assembled nanoparticles [31, 32]. Therefore, due to the above considerations, we designed two subunit vaccine candidates using COE conjugated with or without ferritin nanoparticles. The results showed that the COE-ferritin protein was successfully expressed in *E. coli*, and it could be purified using routine methods similar to those for the COE protein. Moreover, the COE-ferritin protein could self-assemble into nanoparticles with a diameter of 10 nm, making it a promising vaccine candidate.

Since severe clinical symptoms and high mortality rates caused by PEDV infection occur mostly in neonatal piglets (under 7 days old), in which the immune system is naïve and immature, lactogenic immunity remains the most effective way to protect neonatal piglets against PEDV-induced disease [33–36]. In this study, we evaluated the immune responses and protective efficacies of our vaccine candidates in neonatal piglets through the immunization of pregnant sows. The results showed that the levels of PEDV-specific IgG, PEDV-specific IgA, and neutralizing antibodies induced in sows vaccinated with COE-ferritin and their piglets were significantly higher than those elicited in sows vaccinated with COE and their offspring. Moreover, all piglets born to COE-ferritin-vaccinated sows were protected from death and showed considerably reduced clinical symptoms, such as body weight loss, diarrhea, and vomiting, upon challenge with PEDV. In addition, histological analysis at the experimental endpoint demonstrated that intestinal tissue damage in piglets born to COE-ferritin- vaccinated sows was significantly lower than that in piglets born to COE-vaccinated sows. These findings suggest that the nanoparticle vaccine is capable of inducing effective immune responses that are transferred to piglets, providing them with the necessary immunity to survive the viral challenge.

In conclusion, the PEDV COE-ferritin nanoparticle vaccines produced in *E. coli* induce strong humoral immune responses in pregnant sows, which provide protective lactogenic immunity to their newborn piglets, thereby reducing mortality, clinical symptoms, and fecal shedding through challenge exposure with virulent PEDV. These results indicate that COE-ferritin nanoparticles are promising vaccine candidates against PEDV infection.

## Supplementary Information

**Additional file 1: Identification of the expression forms of COE and COE-ferritin in E. coli by 12% SDS-PAGE.** **A** *E. coli* BL21 pET28a-COE. **B** *E. coli* BL21 pET28a-COE-ferritin. lane M, protein markers; lane 1, before induction; lane 2, after induction; lane 3, cell lysate supernatant; lane 4, cell lysate precipitates.

**Additional file 2:** **TEM images of the COE proteins.** Scale bar: 50 nm.

## Data Availability

The data presented in this manuscript are available through the corresponding author upon reasonable request.
