# Supplementary figures and images for: Ferritin-based nanoparticle vaccine protects neonatal piglets against porcine epidemic diarrhea virus challenge following immunization of pregnant sows
Source: Vet Res. 2025 Jul 7;56:140. doi: 10.1186/s13567-025-01542-8 (PMC12235928; doi:10.1186/s13567-025-01542-8)

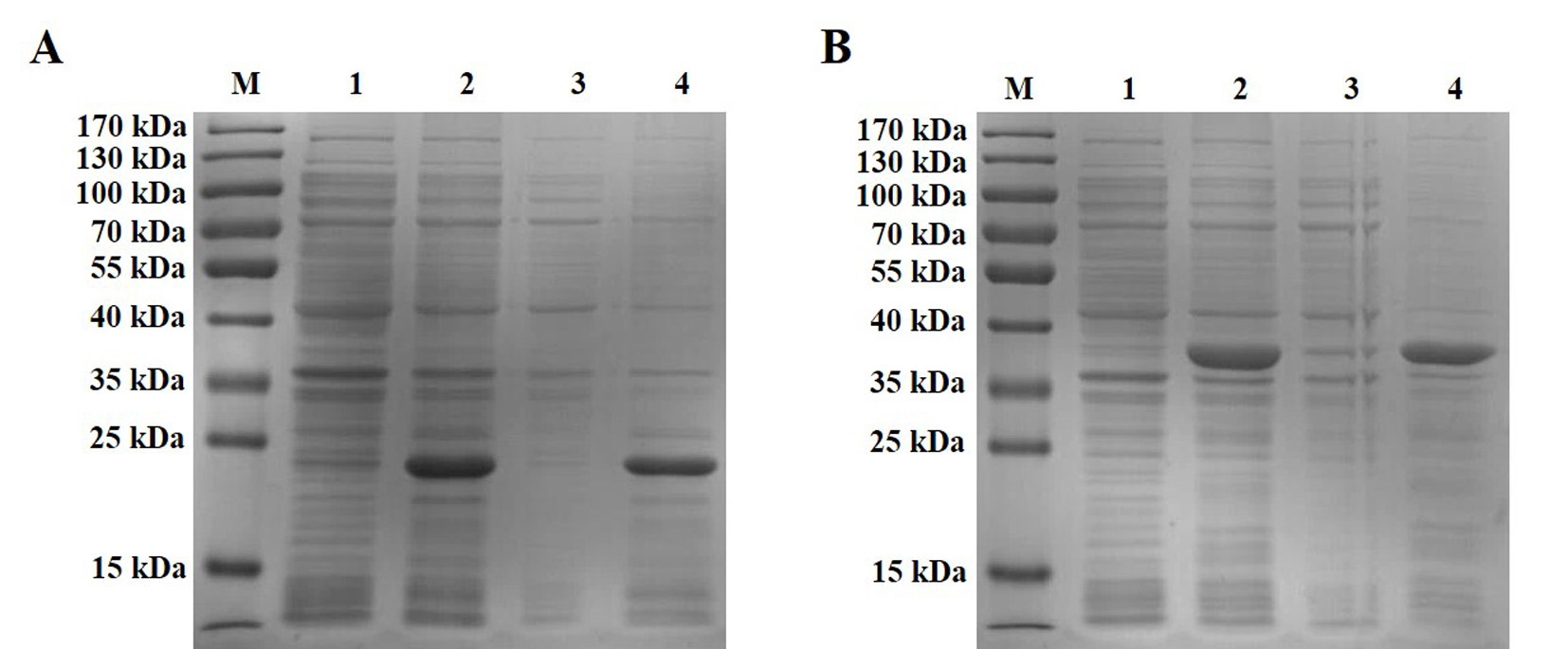

Supplement: Supplementary file 1 — Additional file 1: Identification of the expression forms of COE and COE-ferritin in E. coli by 12% SDS-PAGE. A E. coli BL21 pET28a-COE. B E. coli BL21 pET28a-COE-ferritin. lane M, protein markers; lane 1, before induction; lane 2, after induction; lane 3, cell lysate supernatant; lane 4, cell lysate precipitates. [file 13567_2025_1542_MOESM1_ESM.jpg]

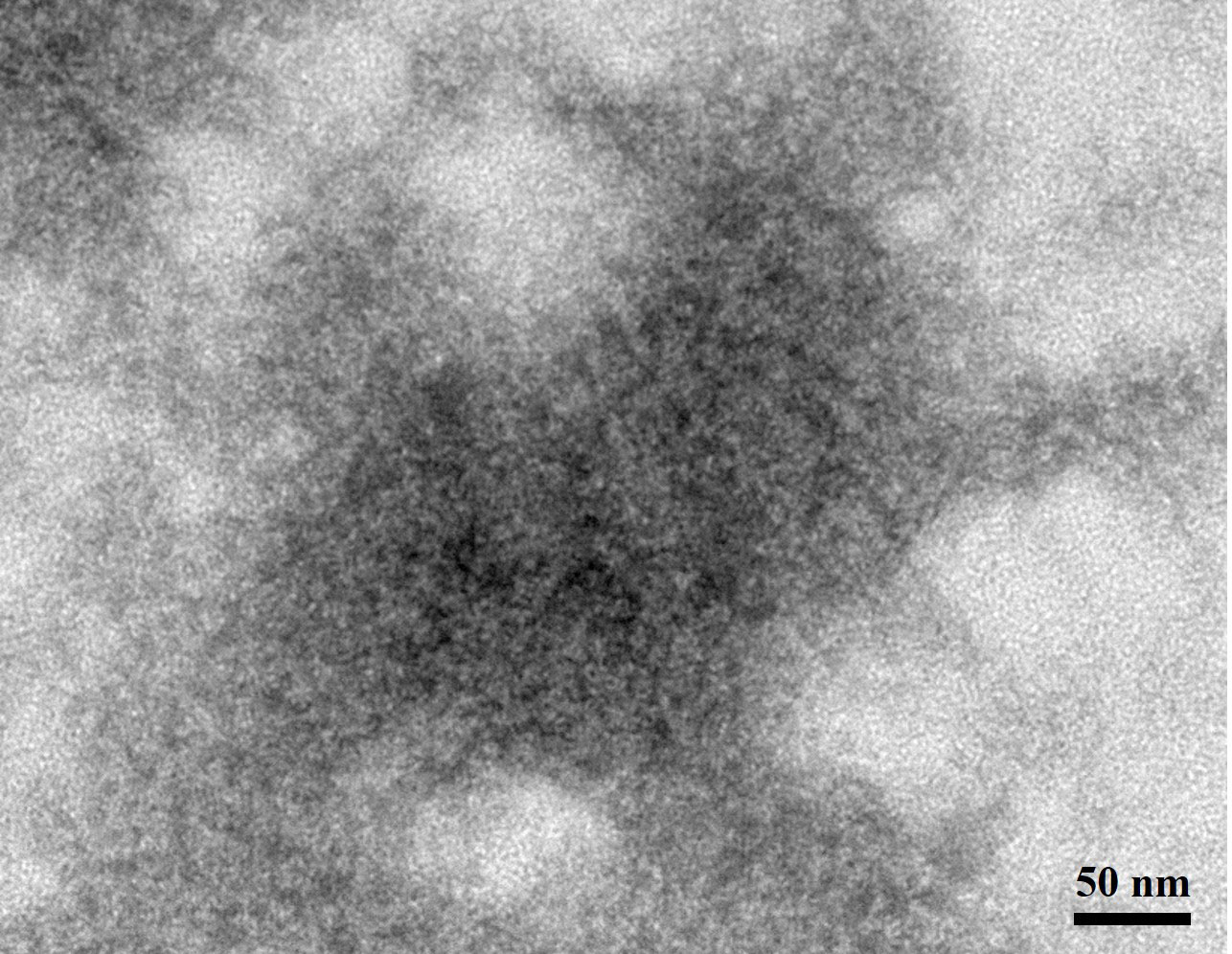

Supplement: Supplementary file 2 — Additional file 2: TEM images of the COE proteins. Scale bar: 50 nm. [file 13567_2025_1542_MOESM2_ESM.jpg]
